# New perspectives on the ligands and function of the killer cell immunoglobulin-like receptor KIR3DL2 in health and disease

**DOI:** 10.3389/fimmu.2012.00339

**Published:** 2012-11-16

**Authors:** Jacqueline Shaw, Simon Kollnberger

**Affiliations:** Nuffield Department of Orthopaedics, Rheumatology and Musculoskeletal Science, Botnar Research Centre, Oxford UniversityOxford, UK

**Keywords:** ankylosing spondylitis, B272, CpG DNA, HLA-A11, HLA-A3, HLA-B27, KIR3DL2, Sézary’s syndrome

## Abstract

KIR3DL2/CD158k/p140 is a three domain killer cell immunoglobulin-like receptor incorporating cytoplasmic immunoreceptor tyrosine inhibitory motifs, expressed as a disulphide-bonded dimer. KIR3DL2 is a framework gene within the KIR locus and is highly polymorphic, with 62 allelic variants possibly coding for protein reported. KIR3DL2 binds to HLA-A3 and -A11 in a peptide-dependent fashion and to B27 free heavy chain forms. In addition, KIR3DL2 can also function as an innate immune receptor for delivery of CpG DNA to TLR9 in NK cells. The increased levels of expression of KIR3DL2 compared with other KIR expressed by T cell subsets in healthy individuals suggest it may function as a default KIR receptor. KIR3DL2-expressing natural killer (NK) cells and IL17 secreting CD4 T cells have been implicated in the pathogenesis of ankylosing spondylitis. Moreover, KIR3DL2 expression delineates circulating and cutaneous lymphoma T cells in Sézary’s syndrome. Here we discuss how the unique molecular attributes of KIR3DL2 impact on its function on NK and T cells and how this may relate to its role in disease.

## INTRODUCTION

KIR3DL2 has the CD designation CD158k. KIR3DL2 has been variously referred to as NKAT4, NKAT4a, NKAT4b, and cl-5. The extracellular ligand-binding regions of the killer cell immunoglobulin-like receptor (KIR) family of which KIR3DL2 is a member are arranged in domains with an immunoglobulin-fold like (Ig) structure. KIR are classified based upon how many extracellular C2-type immunoglobulin-like domains they have (*2D* for two domains, *3D* for three domains) and by the length of their cytoplasmic domain (*L* for long-tailed receptors and *S* for short ones; Marsh et al., 2003).

## GENE ORGANIZATION AND ALLELIC VARIATION

The gene for KIR3DL2 is a framework gene within the KIR gene locus. KIR3DL2 nucleotide sequences are the longest of all KIR genes spanning 16,256 bp in full genomic sequences and coding for a 1,368 bp cDNA (reviewed in the IPD-KIR database: http://www.ebi.ac.uk/ipd/kir). The genomic sequence comprises many putative regulatory elements, some of which are unique to the KIR3DL2 gene, suggesting transcriptional regulation distinct from other KIR genes. These include putative binding sites for RUNX, c-myc, p53, and NFκB transcription factors (Trowsdale et al., 2001; Cichocki et al., 2009). In addition there are also KIR3DL2 allelic variants in non-coding DNA which may have effects on gene transcription.

## KIR3DL2 PROTEIN STRUCTURE

KIR3DL2, is expressed as a protein with signal peptide, sequential D0, D1, and D2 immunoglobulin-like domains, stem and cytoplasmic tail regions. Similar to other three domain KIRs, the KIR3DL2 gene incorporates nine exons. Exons 1–2 encode for signal peptide. The D0, D1, and D2 immunoglobulin-like domains, stem, transmembrane, and cytoplasmic tail regions are encoded by exons 3–5, 6, 7, and 8–9, respectively (Colonna and Samaridis, 1995). The D0, D1, D2 domains, stem, and transmembrane regions of KIR3DL2 proteins are 96, 102, 98, 24, and 20 amino acids in length, respectively. The cytoplasmic tail of KIR3DL2 is the longest of all KIR3D proteins incorporating 96 amino acids and contains two regulatory immunoreceptor tyrosine inhibitory motifs (ITIMs).

KIR3DL2*001 is unique among KIR in being expressed as a 140 kDa dimer (p140, Pende et al., 1996). There are two unpaired cysteines in the stem region at positions 302 and 336 which are not in the D0, D1, and D2 Ig-like domains and which may be involved in disulphide-bonded dimerization.

Eighty four allelic variants of KIR3DL2 have been reported of which 62 encode for potential proteins. The relative expression levels of these different alleles is unknown. Alleles differ by amino acid substitutions. 48 of the allelic variants differ in amino acid sequence in the D0 domain or in the region between the D0 and D1 domains. Whilst the D0 domains of KIR3DL1 and KIR3DS1 are similar, the sequence of KIR3DL2 differs significantly in this region. This strongly suggests that the KIR3DL2 D0 domain has an important role to play in binding to ligand.

## REGULATION OF KIR3DL2 EXPRESSION

A mean of 24.3% [interquartile range (IQR): 18.7–27.7] natural killer (NK) cells express KIR3DL2 in healthy individuals (Chan et al., 2005). These levels are similar to the proportions of NK cells in healthy individuals that express other KIR for which antibodies are available such as KIR3DL1.

KIR3DL2 is expressed on a higher proportion of CD4 and CD8 T cells than other KIR (Chan et al., 2005). We have shown that a mean of 9% (IQR: 4.6–13.7) peripheral blood CD8 T cells and 5% CD4 T cells (IQR: 1.8–6) express KIR3DL2 compared to 0.5 and 1% of CD4 and CD8 T expressing other KIR, respectively. This suggests that KIR3DL2 expression may be less tightly controlled than other KIR. KIR3DL2 expression is enriched on memory CD45RO^+^CD28^-^CCR7^-^CD62L^-^ T cells (Chan et al., 2005). A higher proportion of CD28^-^CD4 T cells express KIR3DL2 than the related KIR3DL1 in both healthy controls and spondyloarthritis (SpA) patients (Chan et al., 2005). CD28^-^CD4 T cells have been implicated in the pathogenesis of both rheumatoid arthritis and SpA (Warrington et al., 2001; Chan et al., 2005; Raffeiner et al., 2005).

Promoter hypomethylation and an active histone signature in KIR-expressing cells are associated with KIR3DL2 gene transcriptional activity but factors which specifically control expression are unclear (Chan et al., 2003; Santourlidis et al., 2008; Zhang et al., 2011).

## HLA CLASS 1 LIGANDS FOR KIR3DL2

NKAT4 or KIR3DL2*001 was originally shown to bind specifically to HLA-A3 and -A11 but not -A1, -A2, and -A24 (Wagtmann et al., 1995; Dohring et al., 1996; Pende et al., 1996). It was later shown that KIR3DL2*001 binding to tetramerized peptide MHC (pMHC) complexes of HLA-A3 and -A11 was critically dependent on the sequence of complexed viral peptide, with the RLRAEAQVK EBV/EBNA3A (603–611) epitope promoting interaction (Hansasuta et al., 2004). As with other KIR-HLA class 1 interactions positions 7 and 8 of complexed peptide were shown to be critical for promoting pMHC complex HLA-A3 and -A11 binding to KIR3DL2. The exact role of bound peptide in pMHC complex recognition by three domain KIRs remains unclear. KIR binding to class 1 distinguishes self from lack of self as may happen when class 1 is down-regulated by virus infection.

Studies on interactions of two domain KIRs with pMHC have shown that KIR-expressing NK cells respond more readily to changes in peptide than to changes in HLA class 1 expression (Fadda et al., 2010). Tumors and viruses do not always necessarily down-regulate HLA class 1 substantially but may change peptide repertoire. Under these circumstances peptide selectivity confers NK cells with an additional recognition mechanism. KIR inhibition of NK function by pMHC is highly sensitive to the presence of antagonist peptides which do not induce inhibition (Fadda et al., 2010). The effect of antagonist peptides in uncoupling KIR clustering from NK inhibition dominates over effects of agonist peptides which promote inhibition. This could enable NK cells to discriminate and respond to small changes in peptide repertoire bound by HLA class 1 at the cell surface during infection.

In NK development strong class 1 ligands for KIR promote or “license” NK cells with better effector function. Most of the identified peptides which promote binding to three domain KIRs are from pathogens and few self peptides which promote strong binding have been identified (Kollnberger et al., 2007). KIR3DL2-expressing NK cells in HLA-A3^+^ individuals have poor effector function against target cells (Fauriat et al., 2008). This suggests that it is unlikely that self peptides which promote binding of pMHC complexes of HLA-A3 to KIR3DL2 are naturally more abundant.

Residues in the D1 domain of KIR3DL2 are probably critical for recognition of HLA-A11 and -A3 since gene conversion has generated KIR2DS4 which binds HLA-A11 but has reduced specificity for HLA-C (Graef et al., 2009).

HLA-B27 (B27) is expressed at the cell surface of antigen presenting cells (APCs) as both β2m-associated and free heavy chain (FHC) species (Kollnberger et al., 2002, Payeli et al., 2012; Bird et al., 2003). B27 has a reactive cysteine at position 67 which results in an increased propensity to form FHC species including dimers at the cell surface. We have shown that KIR3DL2 binds to B27 FHC forms including dimers (Kollnberger et al., 2002). In transfected cells, cell surface B27 heavy chain dimers and other FHC species form from recycling β2m-associated B27 in an endosomal compartment (Bird et al., 2003). Formation of B27 FHC is increased when β2m-associated B27 is suboptimally loaded with peptide (Bird et al., 2003). The extent to which pMHC stability and recycling contribute to formation of B27 FHC species *in vivo* is unknown. B27 heavy chain dimer tetramers but not β2m-associated B27 tetramers bind to KIR3DL2 (Kollnberger et al., 2002, 2007). By contrast, both B27 heavy chain dimers and β2m-associated B27 bind to KIR3DL1 (Kollnberger et al., 2002, 2007). KIR3DL2 ligation by B27 FHC inhibits leukocyte production of IFNγ and promotes NK and T cell survival (Chan et al., 2005; Bowness et al., 2011). Moreover B27 FHC stimulate IL2 production by KIR3DL2CD3ε-transduced Jurkat reporter T cells (Payeli et al., 2012). KIR3DL2 interaction with HLA-B27 FHC forms is inhibited by B27-dimer specific antibodies (Payeli et al., 2012). By contrast with KIR binding to classical HLA class 1 which depends on the sequence of complexed peptide, KIR3DL2 binding to B27 heavy chain forms is independent of the sequence of bound peptide (Kollnberger et al., 2007). By contrast, it is unclear whether B27 FHC interact functionally with KIR3DL1.

It is unknown whether KIR3DL2 interacts functionally with ligand as a dimer or monomer. Since there is no polymorphic variation in this region it is plausible that dimerization is important for receptor function. The inhibition of KIR3DL2 interaction with HLA-B27 by the heavy chain-specific MAb HC10 suggests that this particular KIR may recognize common shared features of HLA class 1 heavy chains (Kollnberger et al., 2007). KIR3DL2 dimerization could increase the avidity of interaction with B27 heavy chains. KIR3DL1 binds more strongly to β2m-associated HLA-B27 tetramers than B27 dimer tetramers (Kollnberger et al., 2007). Weaker interaction of KIR3DL1 with B27 FHC compared to KIR3DL2 could offer one possible explanation for why there is no clear association of KIR3DL1 with SpA. KIR3DL1 has also been shown to bind to common shared features of HLA class 1. The recent structure of KIR3DL1 in complex with HLA-B57 suggests that the D0 domain may bind to more conserved regions of HLA class 1 whereas the D1 and D2 domains provide allelic and peptide-specific contacts (Vivian et al., 2011). The D0 domain acts to enhance KIR3DL1*001 binding to HLA class 1. Phe9, Trp13, His 29, Phe34 amino acids in the D0 domain of KIR3DL1*001 bind loops formed by amino acids 14–18 and 88–92 of HLA-B*5701. These loops are highly conserved between different HLA class 1 alleles. By analogy the D0 domain of KIR3DL2 could have a role to play in recognition of HLA-B27 and other HLA class 1 heavy chains. Notably in KIR3DL2 the D0Trp13 is substituted by a histidine. The relative contributions of these and other residues to binding of KIR3DL2 to B27 FHC forms and other HLA class 1 remain to be determined.

We propose that multimeric forms of B27 heavy chains which include B27 dimers interact more strongly with KIR3DL2 and other immune receptors than other HLA class 1. We have shown that multimeric FHC forms and heavy chain dimers of B27 bind more strongly to the leukocyte immunoglobulin-like receptor LILRB2 than β2m-associated HLA-B27 and other HLA class 1 (Giles et al., 2012). Stronger anti-apoptotic and functional effects resulting from B27 FHC forms binding to KIR3DL2 compared with other HLA class 1 ligands could explain why HLA-B27 and not other HLA class 1 is associated with SpA.

## KIR3DL2 FUNCTION

Although KIR3DL2 ligation on NK cells inhibits IFNγ and cytotoxic function, its effects on T cell function are less clear. KIR3DL2 ligation inhibits target cell lysis by the TALL104 cell line (Brando et al., 2005). However, both antibody and HLA class 1 ligation of KIR3DL2 have been reported to have no effect on CD8 T cell cytokine secretion and cytotoxicity under certain circumstances (Pende et al., 1996). This is consistent with the notion that functional effects of KIR on T cells are dependent on the differentiation and/or activation status of cells expressing these receptors. The contribution of allelic variation to KIR3DL2 recognition of different HLA class 1 is unknown. In addition nothing is known about the effect of allelic variation on antibody recognition of KIR3DL2. The large number of protein-encoding polymorphic variants of KIR3DL2 suggests that the list of class 1 ligands for this receptor is by no means exhaustive.

## INNATE LIGANDS FOR KIR3DL2

Natural killer cells express a range of toll-like receptors including TLR3 and TLR9 and are rapidly activated by TLR binding to PAMPs (reviewed in Marcenaro et al., 2011). Binding of poly I:C and CpG-oligodeoxynucleotide (ODN) PAMPs to their respective TLR, TLR-3 and -9 both promotes NK cytokine release and increases cytotoxicity. Cell surface KIR3DL2 is down-modulated by binding to CpG-ODN and subsequently KIR3DL2 transports bound CpG-ODN to endosomes where TLR9 is ligated (Sivori et al., 2010). KIR3DL2 and KIR3DL1 bind to CpG-ODN via their D0 domain. Although both KIR3DL1 and KIR3DL2 bind in this way, KIR3DL2 modulation is particularly strong upon cell exposure to CpG-ODN and KIR3DL2-expressing NK Cells are more responsive to TLR9-mediated activation. This selective effect of CpG-ODN on KIR3DL2 could be related to dimerization of the molecule and/or differences in the cytoplasmic tail compared to the other three domain KIRs.

## KIR3DL2 EXPRESSION IN DISEASE

### THE ROLE OF KIR3DL2 IN SPONDYLOARTHRITIS

HLA-B27 is strongly associated with a group of inflammatory arthritides collectively known as the spondyloarthritides/SpA (Brown et al., 1996). These disorders, characterized by primary inflammation of the sacroiliac joint, include reactive arthritis, early onset enthesitis related arthritis (ERA), psoriatic arthritis and IBD-associated enteropathic arthritis and are typified by ankylosing spondylitis (AS). GWAS studies have identified genes involved in regulation of IL17 production including the transcription factor STAT3 and the IL23 receptor (IL23R) as having important roles in AS (Reveille et al., 2011). IL17 plays an important role in diverse autoimmune disorders including rheumatoid arthritis and SpA (Wendling et al., 2007; Shen et al., 2009; Bowness et al., 2011). IL17 has multiple proinflammatory actions which include stimulating TNFα production and enhancing the recruitment of other proinflammatory leukocytes including neutrophils to the sites of inflammation (Jovanovic et al., 1998; Laan et al., 1999).

We observe increased proportions of NK and CD4 T cells expressing KIR3DL2 in the peripheral blood and peripheral joints of patients with SpA (Chan et al., 2005; Bowness et al., 2011). Patients with B27^+^ juvenile ERA demonstrated increased proportions of CD4 T cells but not NK cells expressing KIR3DL2 compared to B27 negative healthy and disease controls. By contrast, patients with established AS had greater proportions of both NK and CD4 T cells expressing KIR3DL2 compared to B27 negative controls. This suggests that KIR3DL2 expression by CD4 T cells may have a role in initial inflammation in B27-associated arthritis. By contrast, KIR3DL2-expressing NK cell subsets may be involved in perpetuating inflammation. This is also supported by our observations of greater proportions of KIR3DL2-expressing CD4 T cells in patients with B27^+^ reactive arthritis compared to patients with more established disease (Bowness et al., 2011). KIR3DL2^+^ NK cells from patients expressed high levels of perforin and the CD38 activation marker and were enriched for expression of the β7 integrin suggesting a mucosal and possibly gut origin (Chan et al., 2005). Moreover NK cells from patients were more cytotoxic toward targets than cells from controls (Chan et al., 2005). However, the relative contribution of KIR3DL2-expressing and NK cells lacking KIR3DL2 expression to the increased cytotoxicity was not determined. NK cells stimulate chemokine and MMP production by fibroblasts and are themselves sources of chemokines-like IL8 (Raffeiner et al., 2005; Chan et al., 2008; Fauriat et al., 2010). Apart from increased cytotoxicity it is also possible that KIR3DL2^+^ expressing NK cell subsets in B27^+^ SpA patients enhance inflammation through activation of key coordinating cells such as fibroblasts.

We and other authors have also shown increased expression of HLA class 1 heavy chains and B27 heavy chain dimers on the surface of B27^+^ AS patient leukocytes (; Tsai et al., 2002; Raine et al., 2006). We propose that expansion of KIR3DL2-expressing NK and CD4 T cells in AS patients is driven by higher avidity interactions with B27 FHCs formed in disease. Stronger interactions with KIR3DL2 could result either from B27 FHC forms binding more strongly or higher levels of expression of B27 ligands compared to other HLA class 1.

Increased expression of HC10-reactive heavy chains has also been associated with psoriatic arthritis (Lan et al., 2004). Notably both B27^+^ and B27^-^ psoriatic arthritis patients demonstrated increased expression of heavy chains on peripheral blood monocytes compared to controls, with B27^-^ patients showing significantly higher levels of expression compared to B27^+^ (Lan et al., 2004). Although this hypothesis is attractive, direct evidence for the physiological relevance of KIR interactions with B27 and other HLA class 1 heavy chains *in vivo* remains to be determined. If KIR3DL2-expressing leukocytes are also involved in psoriatic arthritis, it is possible that interactions with other class 1 heavy chains could have a similar effect to B27 in promoting the survival and differentiation of inflammatory leukocytes in these disorders.

KIR3DL2-expressing CD4 T cells are enriched for expression of Th17 phenotypic markers and particularly IL23R expression (Bowness et al., 2011). Notably, although comprising just 15% of all peripheral blood CD4 T cells in SpA, KIR3DL2^+^CD4 T accounted for 60% of all IL23R-expressing CD4 T cells. KIR3DL2-expressing CD4 T cells are also enriched for IL23R expression in healthy B27-controls where they constituted 30% of all IL23R-expressing CD4 T cells. Moreover, KIR3DL2^+^CD4 T cells accounted for the majority of the increase in IL17 production by peripheral blood CD4 T cells in SpA patients compared to healthy and disease controls (Bowness et al., 2011).

The greater expression of KIR3DL2 on CD4 T cells compared to other KIR allowed us to directly FACS sort this population from peripheral blood and measure cytokine production by short term cultures stimulated with anti-CD3, anti-CD28, and anti-CD2. KIR3DL2 CD4 T cells from SpA patients consistently produced more IL17 than the same subset isolated from controls. IL17 production by KIR3DL2 CD4 T cell subsets from patients and controls was further promoted by stimulation in the presence of the Th17 cytokines IL23 and IL1 (Bowness et al., 2011). However, stimulation with Th17 cytokines promoted a greater increase in IL17 production by the KIR3DL2 CD4 T cell subset from SpA patients compared to increases in IL17 production by the same subset in controls.

The observation of production of IL17 by KIR-expressing CD4 T cells contradicts previous observations where KIR-expressing CD4 T cells were shown to produce only small levels of Th17 cytokines (van Bergen et al., 2004). It is possible that previous work with KIR-expressing CD4 T cell lines and clones selected for outgrowth of Th1 cells and IL17 producing cells could have been missed.

We did not observe significant increases in IL23R expression on CD4 T cells from SpA patients lacking KIR3DL2 expression (Bowness et al., 2011). We consistently observe expansion of KIR3DL2-expressing CD4 T cells in B27^+^ SpA patients and B27^+^ but not B27-healthy controls. The increase in KIR3DL2 expression on CD4 T cells in the peripheral blood of B27^+^ healthy controls is intermediate between healthy B27^-^ controls and B27^+^ SpA patients (Bowness et al., 2011). This strongly suggests that B27 interactions with KIR3DL2 have a central role to play in SpA. KIR3DL2 interactions with B27 could also have a role to play in other diseases where HLA-B27 has been shown to play a role. In addition, the level of surface expression of HLA-B27 may have a key role in determining progression toward arthritis. Indeed increased levels of both β2m-associated and FHC forms of B27 have been associated with disease (Cauli et al., 2002; Tsai et al., 2002; Raine et al., 2006).

We have shown that cell lines expressing HLA-B27 FHC promote the survival of KIR3DL2 CD4 T cells secreting IL17 (Bowness et al., 2011). The Th1 cytokine IFNγ negatively regulates IL17 production both by directly inhibiting Th17 cell production and also inhibiting IL23 production by APCs (Mills, 2008; Sheikh et al., 2010). In addition to promoting leukocyte survival, KIR3DL2 ligation by B27 could also promote disease by limiting production of Th1 cytokines, thus skewing leukocytes toward production of IL17. If B27 FHC are indeed stronger ligands for KIR3DL2 than other β2m-associated HLAclass 1, stronger binding of B27 to KIR3DL2 could have a more pronounced effect on inhibition of IFNγ production than other HLA class 1.

KIR3DL2 ligation by B27 also promotes the survival of NK cells and inhibits their production of IFN-γ (Chan et al., 2005). B27 interactions with KIR3DL2 could thus have a role in promoting the expansion of the increased proportions of KIR3DL2^+^ NK cells observed in B27^+^ patients with SpA. The demonstration of a functional interaction between KIR3DL2 and HLA-B27 raises the possibility that this interaction could be involved in the development of NK effector function or licensing in SpA and other diseases with B27 involvement.

No specific alleles of KIR3DL2 have been associated with SpA (Harvey et al., 2009). These studies do not exclude the possibility that a rare allelic variant of KIR3DL2 is associated with disease. We found a similar effect of B27-expressing APC in promoting the survival of KIR3DL2-expressing CD4 T cells from a wide range of control and SpA subjects. This suggests that KIR3DL2 polymorphism has little effect on the interaction with B27 FHC, although KIR3DL2 allelic variation could affect the strength of interaction.

### THE ROLE OF KIR3DL2 IN SÉZARY’S SYNDROME

Apart from SpA, KIR3DL2 expression delineates both circulating and infiltrating T cell lymphoma cells in Sézary’s syndrome (SS; Musette et al., 2003; Poszepczynska-Guigne et al., 2004). SS is not associated with HLA-B27 and the ligand/ligands for KIR3DL2 in this disease are unknown. SS is an erythrodermic, aggressive, cutaneous T-cell lymphoma characterized by a malignant T-cell clone that localizes in the blood and skin. Expansion of SS T cells could be driven by innate ligands such as bacterial CpG DNA, or KIR3DL2 expression could occur as a secondary consequence of T cell transformation. The demonstration of expansions of non-lymphoma T cells expressing KIR3DL2 in SS patients suggests that the latter possibility is unlikely. SS T cells and KIR3DL2-expressing CD4 T cells in SS patients have a restricted Vβ TCR repertoire suggesting antigen-driven expansion of these cells (Ortonne et al., 2006). KIR3DL2 expression has been proposed to be induced on SS T cells by bacterial exposure. KIR3DL2 expression has also been reported to be up-regulated on T cells in adult T cell leukemia which is strongly associated with HTLV-1 infection and associated infective dermatitis (Obama et al., 2007).

T cells from patients with SS demonstrate constitutive phosphorylation of pSTAT3 and secrete IL17 (Ciree et al., 2004; van der Fits et al., 2012). KIR3DL2 ligation by an as yet uncharacterized ligand may promote expansion of T cells in SS. Candidate ligands could be CpG DNA or HLA class 1 heavy chains. Alternatively, increased KIR3DL2 expression could result from T cell activation independently of binding to ligand. SS T cells may also have a role in neutrophil recruitment to inflammatory sites in the skin in SS as neutrophil recruitment correlates with IL17 production by these cells. Although IL17 itself is not essential, IL21 produced by SS T cells has an important role to play in the maintenance of this subset (van der Fits et al., 2012).

KIR3DL2 CD4 T cells in SpA have a highly activated phenotype suggesting that expression may be induced by chronic T cell activation (Chan et al., 2005). B27^+^ reactive arthritis is associated with gram-negative intracellular bacterial infection. Evidence of subclinical gut inflammation in SpA patients and the association of HLA-B27 with inflammatory bowel disease also link SpA with bacterial infection (Ciccia et al., 2009; Brown, 2011). One possibility is that KIR3DL2 expression in SpA and SS is linked to bacterial activation of leukocytes.

## CONCLUDING REMARKS

**FIGURE 1:**
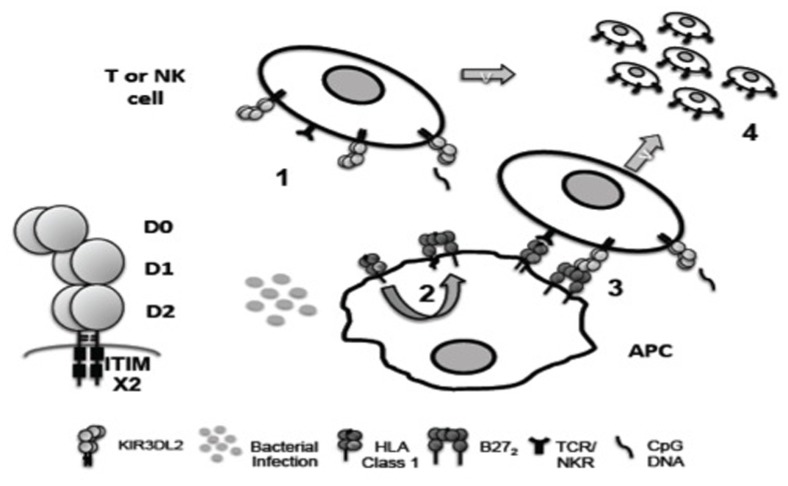
**Hypothetical role of KIR3DL2 ligand interactions in inflammation.** KIR3DL2 is expressed as a disulphide-bonded dimer with D0, D1, D2 immunoglobulin-like domain organization and two cytoplasmic ITIM motifs. (1) KIR3DL2 expression is induced by T cell activation and/or NK differentiation. (2) Bacterial infection may promote formation of B27 dimers (B27_2_) and free heavy chains (FHC) by enhanced recycling of β2m-associated HLA class 1 by antigen presenting cells (APC). (3) B27 FHC including B27_2_ could bind more strongly to KIR3DL2 than β2m-associated HLA class 1. B27 FHC are predicted to bind to KIR3DL2 via the D0 domain. (4) KIR3DL2 binding to ligand inhibits activation induced cell death driven by TCR/NK receptor ligand interactions, promoting the survival of pathogenic Th17 and NK cell subsets in disease. The D0 domain of KIR3DL2 also binds bacterial CpG DNA. CpG DNA internalized by KIR3DL2 could costimulate NK and T cell via TLR9 ligation (1 and 4).

KIR3DL2 is a highly polymorphic framework KIR gene. **Figure 1** summarizes how KIR3DL2 expression and ligation may contribute to immune regulation and inflammation. Differences in potential transcription binding sites between KIR3DL2 and other KIR genes and the large size of the KIR3DL2 gene locus suggest unique transcriptional control. KIR3DL2 ligands include HLA-A3 and -A11, HLA-B27 FHC dimers and other FHCs and CpG-ODN. The influence of KIR3DL2 dimerization on ligand binding remains to be determined. KIR3DL2 expression is up-regulated on activated CD4 T cells and NK cells in SpA and circulating and T cell lymphoma cells in SS. Since both disorders are associated with bacterial infection, it is possible that bacterial exposure in general may have a role in promoting the expansion of KIR3DL2-expressing T cells. The enrichment of expression of Th17 phenotypic markers and production of IL17 by peripheral KIR3DL2-expressing CD4 T cells suggests a unique role for KIR3DL2 expression in the differentiation of Th17 cells.

## Conflict of Interest Statement

The authors declare that the research was conducted in the absence of any commercial or financial relationships that could be construed as a potential conflict of interest.

## References

[B1] Bird L. A. Peh C. A. Kollnberger S. Elliott T. McMichael A. J. Bowness P. (2003). Lymphoblastoid cells express HLA-B27 homodimers both intracellularly and at the cell surface following endosomal recycling. *Eur. J. Immunol.* 33 748–759 12616495 10.1002/eji.200323678

[B2] Bowness P. Ridley A. Shaw J. Chan A. Wong Baeza I. Fleming M. (2011). Th17 cells expressing KIR3DL2+ and responsive to HLA-B27 homodimers are increased in Ankylosing Spondylitis. *J. Immunol.* 186 2672–2680 21248258 10.4049/jimmunol.1002653 PMC3210561

[B3] Brando C. Mukhopadhyay S. Kovacs E. Medina R. Patel P. Catina T. L. (2005). Receptors and lytic mediators regulating anti-tumor activity by the leukemic killer T cell line TALL-104. *J. Leukoc. Biol.* 78 359–371 15937142 10.1189/jlb.0604360

[B4] Brown M. A. (2011). Progress in the genetics of ankylosing spondylitis. Brief. Funct. Genomics 10 249–257 21965815 10.1093/bfgp/elr023

[B5] Brown M. A. Pile K. D. Kennedy L. G. Calin A. Darke C. Bell J. (1996). HLA class I associations of ankylosing spondylitis in the white population in the United Kingdom. *Ann. Rheum. Dis.* 55 268–270 8733445 10.1136/ard.55.4.268 PMC1010149

[B6] Cauli A. Dessole G. Nurchis P. P. Vacca A. Mameli A. Garau P. (2002). The role of HLA-B27 molecules in the pathogenesis of ankylosing spondylitis. *Reumatismo* 54 266–271 12404037 10.4081/reumatismo.2002.266

[B7] Chan A. T. Kollnberger S. D. Wedderburn L. R. Bowness P. (2005). Expansion and enhanced survival of natural killer cells expressing the killer immunoglobulin-like receptor KIR3DL2 in spondylarthritis. *Arthritis Rheum.* 52 3586–3595 16255049 10.1002/art.21395

[B8] Chan A. Filer A. Parsonage G. Kollnberger S. Gundle R. Buckley C. D. (2008). Mediation of the proinflammatory cytokine response in rheumatoid arthritis and spondylarthritis by interactions between fibroblast-like synoviocytes and natural killer cells. *Arthritis Rheum.* 58 707–717 18311795 10.1002/art.23264

[B9] Chan H. W. Kurago Z. B. Stewart C. A. Wilson M. J. Martin M. P. Mace B. E. (2003). DNA methylation maintains allele-specific KIR gene expression in human natural killer cells. *J. Exp. Med.* 197 245–255 12538663 10.1084/jem.20021127 PMC2193817

[B10] Ciccia F. Bombardieri M. Principato A. Giardina A. Tripodo C. Porcasi R. (2009). Overexpression of interleukin-23, but not interleukin-17, as an immunologic signature of subclinical intestinal inflammation in ankylosing spondylitis. *Arthritis Rheum.* 60 955–965 19333939 10.1002/art.24389

[B11] Cichocki F. Hanson R. J. Lenvik T. Pitt M. McCullar V. Li H. (2009). The transcription factor c-Myc enhances KIR gene transcription through direct binding to an upstream distal promoter element. *Blood* 113 3245–3253 18987359 10.1182/blood-2008-07-166389 PMC2665893

[B12] Ciree A. Michel L. Camilleri-Broet S. Jean Louis F. Oster M. Flageul B. (2004). Expression and activity of IL-17 in cutaneous T-cell lymphomas (mycosis fungoides and Sezary syndrome). *Int. J. Cancer* 112 113–120 15305382 10.1002/ijc.20373

[B13] Colonna M. Samaridis J. (1995). Cloning of immunoglobulin-superfamily members associated with HLA-C and HLA-B recognition by human natural killer cells. *Science* 268 405–408 7716543 10.1126/science.7716543

[B14] Dohring C. Scheidegger D. Samaridis J. Cella M. Colonna M. (1996). A human killer inhibitory receptor specific for HLA-A1,2. *J. Immunol.* 156 3098–3101 8617928

[B15] Fadda L. Borhis G. Ahmed P. Cheent K. Pageon S. V. Cazaly A. (2010). Peptide antagonism as a mechanism for NK cell activation. *Proc. Natl. Acad. Sci. U.S.A.* 107 10160–10165 20439706 10.1073/pnas.0913745107 PMC2890497

[B16] Fauriat C. Andersson S. Bjorklund A. T. Carlsten M. Schaffer M. Bjorkstrom N. K. (2008). Estimation of the size of the alloreactive NK cell repertoire: studies in individuals homozygous for the group A KIR haplotype. *J. Immunol.* 181 6010–6019 18941190 10.4049/jimmunol.181.9.6010

[B17] Fauriat C. Long E. O. Ljunggren H. G. Bryceson Y. T. (2010). Regulation of human NK-cell cytokine and chemokine production by target cell recognition. *Blood* 115 2167–2176 19965656 10.1182/blood-2009-08-238469 PMC2844017

[B18] Giles J. Shaw J. Piper C. Wong-Baeza I. McHugh K. Ridley A. (2012). HLA-B27 homodimers and free heavy chains are stronger ligands for LILRB2 than classical HLA class 1. *J. Immunol*. 188 6184–6193 22593621 10.4049/jimmunol.1102711 PMC3622243

[B19] Graef T. Moesta A. K. Norman P. J. Abi-Rached L. Vago L. Older Aguilar A. M. (2009). KIR2DS4 is a product of gene conversion with KIR3DL2 that introduced specificity for HLA-A*11 while diminishing avidity for HLA-C. *J. Exp. Med.* 206 2557–2572 19858347 10.1084/jem.20091010 PMC2768862

[B20] Hansasuta P. Dong T. Thananchai H. Weekes M. Willberg C. Aldemir H. (2004). Recognition of HLA-A3 and HLA-A11 by KIR3DL2 is peptide-specific. *Eur. J. Immunol.* 34 1673–1679 15162437 10.1002/eji.200425089

[B21] Harvey D. Pointon J. J. Sleator C. Meenagh A. Farrar C. Sun J. Y. (2009). Analysis of killer immunoglobulin-like receptor genes in ankylosing spondylitis. *Ann. Rheum. Dis.* 68 595–598 19019897 10.1136/ard.2008.095927

[B22] Jovanovic D. V. Di Battista J. A. Martel-Pelletier J. Jolicoeur F. C. He Y. Zhang M. (1998). IL-17 stimulates the production and expression of proinflammatory cytokines, IL-beta and TNF-alpha, by human macrophages. *J. Immunol.* 160 3513–3521 9531313

[B24] Kollnberger S. Bird L. Sun M. Y. Retiere C. Braud V. M. McMichael A. (2002). Cell-surface expression and immune receptor recognition of HLA-B27 homodimers. *Arthritis Rheum.* 46 2972–2982 12428240 10.1002/art.10605

[B25] Kollnberger S. Chan A. Sun M. Y. Chen L. Y. Wright C. di Gleria K. (2007). Interaction of HLA-B27 homodimers with KIR3DL1 and KIR3DL2, unlike HLA-B27 heterotrimers, is independent of the sequence of bound peptide. *Eur. J. Immunol.* 37 1313–1322 17407096 10.1002/eji.200635997

[B26] Laan M. Cui Z. H. Hoshino H. Lotvall J. Sjostrand M. Gruenert D. C. (1999). Neutrophil recruitment by human IL-17 via C-X-C chemokine release in the airways. *J. Immunol.* 162 2347–2352 9973514

[B27] Lan C. E. Tsai W. C. Wu C. S. Lu C. L. Yu H. S. (2004). Psoriatic patients with arthropathy show significant expression of free HLA class I heavy chains on circulating monocytes: a potential role in the pathogenesis of psoriatic arthritis. *Br. J. Dermatol.* 151 24–31 15270869 10.1111/j.1365-2133.2004.05890.x

[B28] Marcenaro E. Carlomagno S. Pesce S. Moretta A. Sivori S. (2011). Bridging innate NK cell functions with adaptive immunity. *Adv. Exp. Med. Biol.* 780 45–55 21842364 10.1007/978-1-4419-5632-3_5

[B29] Marsh S. G. Parham P. Dupont B. Geraghty D. E. Trowsdale J. Middleton D. (2003). Killer-cell immunoglobulin-like receptor (KIR) nomenclature report, 2002. *Immunogenetics* 55 220–226 12838378 10.1007/s00251-003-0571-z

[B30] Mills K. H. (2008). Induction, function and regulation of IL-17-producing T cells. *Eur. J. Immunol.* 38 2636–2649 18958872 10.1002/eji.200838535

[B31] Musette P. Michel L. Jean-Louis F. Bagot M. Bensussan A. (2003). Polymorphic expression of CD158k/p140/KIR3DL2 in Sezary patients. *Blood* 101 1203 10.1182/blood-2002-09-2915 12529298

[B32] Obama K. Kubota R. Tara M. Furukawa Y. Osame M. Arimura K. (2007). Killer cell immunoglobulin-like receptor/3DL2 expression in adult T-cell leukaemia. *Br. J. Haematol.* 138 666–667 17686060 10.1111/j.1365-2141.2007.06704.x

[B33] Ortonne N. Huet D. Gaudez C. Marie-Cardine A. Schiavon V. Bagot M. (2006). Significance of circulating T-cell clones in Sézary syndrome. *Blood* 107 4030–4038 16418328 10.1182/blood-2005-10-4239

[B34] Payeli S. K. Kollnberger S. Marroquin Belaunzaran O. Thiel M. McHugh K. Giles J. (2012). Inhibiting HLA-B27 homodimer-driven immune cell inflammation in spondylarthritis. *Arthritis Rheum.* 64 3139–3149 22576154 10.1002/art.34538

[B35] Pende D. Biassoni R. Cantoni C. Verdiani S. Falco M. di Donato C. (1996). The natural killer cell receptor specific for HLA-A allotypes: a novel member of the p58/p70 family of inhibitory receptors that is characterized by three immunoglobulin-like domains and is expressed as a 140-kD disulphide-linked dimer. *J. Exp. Med.* 184 505–518 8760804 10.1084/jem.184.2.505 PMC2192700

[B36] Poszepczynska-Guigne E. Schiavon V. D’Incan M. Echchakir H. Musette P. Ortonne N. (2004). CD158k/KIR3DL2 is a new phenotypic marker of Sezary cells: relevance for the diagnosis and follow-up of Sezary syndrome. *J. Invest. Dermatol.* 122 820–823 15086570 10.1111/j.0022-202X.2004.22326.x

[B37] Raffeiner B. Dejaco C. Duftner C. Kullich W. Goldberger C. Vega S. C. (2005). Between adaptive and innate immunity: TLR4-mediated perforin production by CD28null T-helper cells in ankylosing spondylitis. *Arthritis Res. Ther.* 7 R1412–R1420 16277694 10.1186/ar1840 PMC1297589

[B38] Raine T. Brown D. Bowness P. Hill Gaston J. S. Moffett A. Trowsdale J. (2006). Consistent patterns of expression of HLA class I free heavy chains in healthy individuals and raised expression in spondyloarthropathy patients point to physiological and pathological roles. *Rheumatology (Oxford)* 45 1338–1344 16936330 10.1093/rheumatology/kel305

[B39] Reveille J. D. Sims A. M. Danoy P. Evans D. M. Leo P. Pointon J. J. (2011). Genome-wide association study of ankylosing spondylitis identifies non-MHC susceptibility loci. *Nat. Genet.* 43 761–767 20062062 10.1038/ng.513 PMC3224997

[B40] Santourlidis S. Graffmann N. Christ J. Uhrberg M. (2008). Lineage-specific transition of histone signatures in the killer cell Ig-like receptor locus from hematopoietic progenitor to NK cells. *J. Immunol.* 180 418–425 18097043 10.4049/jimmunol.180.1.418

[B41] Sheikh S. Z. Matsuoka K. Kobayashi T. Li F. Rubinas T. Plevy S. E. (2010). Cutting edge: IFN-gamma is a negative regulator of IL-23 in murine macrophages and experimental colitis. *J. Immunol.* 184 4069–4073 20228197 10.4049/jimmunol.0903600 PMC2956738

[B42] Shen H. Goodall J. C Hill Gaston J. S. (2009). Frequency and phenotype of peripheral blood Th17 cells in ankylosing spondylitis and rheumatoid arthritis. *Arthritis Rheum.* 60 1647–1656 19479869 10.1002/art.24568

[B43] Sivori S. Falco M. Carlomagno S. Romeo E. Soldani C. Bensussan A. (2010). A novel KIR-associated function: evidence that CpG DNA uptake and shuttling to early endosomes is mediated by KIR3DL2. *Blood* 116 1637–1647 20147700 10.1182/blood-2009-12-256586

[B44] Trowsdale J. Barten R. Haude A. Stewart C. A. Beck S. Wilson M. J. (2001). The genomic context of natural killer receptor extended gene families. *Immunol. Rev.* 181 20–38 11513141 10.1034/j.1600-065x.2001.1810102.x

[B45] Tsai W. C. Chen C. J. Yen J. H. Ou T. T. Tsai J. J. Liu C. S. (2002). Free HLA class I heavy chain-carrying monocytes – a potential role in the pathogenesis of spondyloarthropathies. *J. Rheumatol.* 29 966–972 12022359

[B46] van Bergen J. Thompson A. van der Slik A. Ottenhoff T. H. Gussekloo J. Koning F. (2004). Phenotypic and functional characterization of CD4 T cells expressing killer Ig-like receptors. *J. Immunol.* 173 6719–6726 15557164 10.4049/jimmunol.173.11.6719

[B47] van der Fits L. Out-Luiting J. J. van Leeuwen M. A. Samsom J. N. Willemze R. Tensen C. P. (2012). Autocrine IL-21 stimulation is involved in the maintenance of constitutive STAT3 activation in Sezary Syndrome. *J. Investig. Dermatol.* 132 440–447 21938013 10.1038/jid.2011.293

[B48] Vivian J. P. Duncan R. C. Berry R. O’Connor G. M. Reid H. H. Beddoe T. (2011). Killer cell immunoglobulin-like receptor 3DL1-mediated recognition of human leukocyte antigen B. *Nature* 479 401–405 22020283 10.1038/nature10517 PMC3723390

[B49] Wagtmann N. Biassoni R. Cantoni C. Verdiani S. Malnati M. S. Vitale M. (1995). Molecular clones of the p58 NK cell receptor reveal immunoglobulin-related molecules with diversity in both the extra- and intracellular domains. *Immunity* 2 439–449 7749980 10.1016/1074-7613(95)90025-x

[B50] Warrington K. J. Takemura S. Goronzy J. J. Weyand C. M. (2001). CD4+, CD28-T cells in rheumatoid arthritis patients combine features of the innate and adaptive immune systems. *Arthritis Rheum.* 44 13–20 11212151 10.1002/1529-0131(200101)44:1<13::AID-ANR3>3.0.CO;2-6

[B51] Wendling D. Cedoz J.-P. Racadot E. Dumoulin G. (2007). Serum IL-17, BMP-7, and bone turnover markers in patients with ankylosing spondylitis. *Joint Bone Spine* 74 304–305 17369068 10.1016/j.jbspin.2006.11.005

[B52] Zhang L. Dai Y. Wang L. Peng W. Zhang Y. Ou Y. (2011). CpG array analysis of histone H3 lysine 4 trimethylation in peripheral blood mononuclear cells of uremia patients. *DNA Cell Biol.* 30 179–186 21155670 10.1089/dna.2010.1076

